# Changes in the Incidence Rates of Gastrointestinal Diseases Due to the COVID-19 Pandemic in South Korea: A Long-Term Perspective

**DOI:** 10.3390/jpm12071144

**Published:** 2022-07-14

**Authors:** Hyo Geun Choi, Ho Suk Kang, Hyun Lim, Joo-Hee Kim, Ji Hee Kim, Seong-Jin Cho, Eun Sook Nam, Kyueng-Whan Min, Ha Young Park, Nan Young Kim, Mi Jung Kwon

**Affiliations:** 1 Department of Otorhinolaryngology-Head & Neck Surgery, Hallym University Sacred Heart Hospital, Hallym University College of Medicine, Anyang 14068, Korea; pupen@naver.com; 2 Division of Gastroenterology, Department of Internal Medicine, Hallym University Sacred Heart Hospital, Hallym University College of Medicine, Anyang 14068, Korea; hskang76@hallym.or.kr (H.S.K.); hlim77@hallym.or.kr (H.L.); 3 Division of Pulmonary, Allergy, and Critical Care Medicine, Department of Medicine, Hallym University Sacred Heart Hospital, Hallym University College of Medicine, Anyang 14068, Korea; luxjhee@gmail.com; 4 Department of Neurosurgery, Hallym University Sacred Heart Hospital, Hallym University College of Medicine, Anyang 14068, Korea; kimjihee.ns@gmail.com; 5 Department of Pathology, Kangdong Sacred Heart Hospital, Hallym University College of Medicine, Seoul 05355, Korea; apilas@hanmail.net (S.-J.C.); esnam@kdh.or.kr (E.S.N.); 6 Department of Pathology, Hanyang University Guri Hospital, Hanyang University College of Medicine, Guri 11923, Korea; kyueng@gmail.com; 7 Department of Pathology, Busan Paik Hospital, Inje University College of Medicine, Busan 47392, Korea; hy08.park@gmail.com; 8 Hallym Institute of Translational Genomics and Bioinformatics, Hallym University Medical Center, Anyang 14068, Korea; honeyny78@gmail.com; 9 Department of Pathology, Hallym University Sacred Heart Hospital, Hallym University College of Medicine, Anyang 14068, Korea

**Keywords:** COVID-19, gastrointestinal disease, epidemiology, incidence rate

## Abstract

We investigated whether the coronavirus disease 2019 (COVID-19) pandemic, in conjunction with public health measures, influenced the incidence of gastrointestinal diseases according to age and sex during the pandemic. Changes in the monthly incidence rates (January 2018 to June 2021) of common gastrointestinal diseases were assessed using data from the Korean National Health Insurance Service by comparing the data of two periods: before COVID-19 (January 2018–February 2020) and during COVID-19 (March 2020–June 2021). The Mann–Whitney *U* test and Levene’s test were used to compare the differences in the incidences before and during the pandemic. In the pandemic period, compared to in the pre-COVID-19 period, the incidence rates of ulcerative colitis, Crohn’s disease, cholelithiasis, and esophageal reflux significantly increased, whereas those of infective enteritis and irritable bowel syndrome decreased, regardless of age or sex. There were no significant changes in the incidence rates of pancreatitis, acute appendicitis, liver cirrhosis, and hemorrhoids. No seasonal variations in gastrointestinal disease occurrence were observed. In conclusion, the COVID-19 pandemic may have had unprecedented and long-term impacts on the epidemiology of gastrointestinal disease. These changes may indicate a substantial future burden on healthcare resources during the recovery phase of the pandemic and thereafter.

## 1. Introduction

The coronavirus disease 2019 (COVID-19) pandemic has led to a shift in the patterns of incidences of various diseases, and a paradigm shift in healthcare systems over the past 2 years [1,2]. A wide range of public health interventions, including both non-pharmaceutical (e.g., hand washing, social distancing, face mask use, voluntary quarantine, travel restrictions, limiting nonessential activities, school closures, and shutting down borders) and pharmaceutical (e.g., ventilators and vaccines) measures have been enforced worldwide against the COVID-19 pandemic [3], of which the former may inhibit human-to-human infections by avoiding the transmission of aerosols that are emitted by infected patients [4]. These measures have led to unprecedented changes in behavioral patterns, as well as the occurrence of other infectious diseases [5]. For example, previous studies have shown a significant decline in the incidence of upper and lower respiratory tract infections such as seasonal influenza, and some non-infectious diseases in pediatric populations [5,6,7,8,9,10]. However, there are a lack of national-level data on yearly or seasonal changes in the incidence rates of gastrointestinal (GI) diseases, which are commonly encountered in clinical settings.

The number of endoscopic and surgical procedures for GI diseases declined by 50–90% during the COVID-19 pandemic [1,2,11,12], with an overall operation cancellation rate of 72% for benign GI diseases [12]. As compared with corresponding durations in 2018 and 2019, in 2020–2022, regular follow-up visits for GI diseases were interrupted or delayed by the pandemic [1,2,13]. The spread of COVID-19 has been impeded due to the enforcement of preventive hygiene policies such as mask wearing, hand washing, and social distancing [5]. These lifestyle changes have also drastically reduced the incidence of other infectious diseases [5], such as GI viral infections that are transmitted through the fecal-to-oral route or direct contact between people [14]. Hence, the COVID-19 pandemic may have had a major influence on the occurrence and management of other GI diseases [6]. Since GI symptoms are also observed in patients with COVID-19 and have been associated with severe COVID-19 disease [15], healthcare providers should promptly differentiate between COVID-19 and non-COVID-19 GI diseases, keeping in mind the prevalence of GI diseases. Nevertheless, there is a lack of studies on the incidence trends of non-COVID-19 GI diseases and the influence of age, sex, and seasonal variation on the same during the national pandemic crisis. In anticipation of the recovery phase of the pandemic and the subsequent post-pandemic era, it is important for healthcare providers to develop preventive programs than can address a potentially significant shift in the incidence of GI diseases.

The present study analyzed data from the nationwide Korean National Health Insurance Service database to determine the changes in the incidence rates of GI diseases due to the COVID-19 pandemic, as well as the influence of seasonal variation, age, and sex. We hypothesized that the incidence rates of both infectious and non-infectious GI diseases were impacted by the COVID-19 pandemic. This study highlights the importance of monitoring GI disease incidence during the COVID-19 pandemic and the need for appropriate planning to address the anticipated challenges in prevention and treatment during the post-pandemic period.

## 2. Materials and Methods

### 2.1. Ethics

The study protocol was approved by the ethics committee of Hallym University (2021-11-004). The requirement for written informed consent was waived by the institutional review board.

### 2.2. Participants and Data Collection

South Korea’s population of over 50 million is under the National Health Insurance (NHI) program. The NHI collects medical information from facilities ranging from primary clinics to tertiary hospitals in one unified database. These data are publicly available for national-level research on disease epidemiology. As the first COVID-19 patients in South Korea were identified on 20 January 2020 and disease prevention and infection control measures were started on the national level in March 2020, we compared the following two periods in the present study: January 2018–February 2020 (before COVID-19) and March 2020–June 2021 (during COVID-19).

We evaluated the monthly incidence rates of the top 10 GI diseases that were common at medical institutions from January 2018 through to June 2021. The incidences of those diseases were investigated using the medical histories of patients’ who visited the clinics for each GI disease. The diagnosis of GI diseases was based on the following International Classification of Diseases-10 codes: cholelithiasis (K80); pancreatitis (K85, K860, K861); infective enteritis (A04, A05, A08, A09); Crohn’s disease (K50); ulcerative colitis (K51); acute appendicitis (K35, K36, K37); liver cirrhosis (K74, K702, K703); esophageal reflux (K21); hemorrhoids including perianal venous thrombosis (K64); and irritable bowel syndrome including other functional intestinal disorders (K58, K59). The diagnostic codes are registered by physicians. Since the patients were identified with a unique resident registration number in the NHI that covered both hospitals and primary clinics, none of the disease incidence data were duplicated.

### 2.3. Statistical Analysis

Differences in the mean incidence rates of the diseases before and during the COVID-19 pandemic were compared using the Mann–Whitney *U* test for nonparametric values. The differences in the variances of medical visits for the diseases before and during the COVID-19 pandemic were compared using Levene’s test for nonparametric values [16]. Subgroup analyses were performed for age (0–19, 20–59, and 60+ years old) and sex. Two-tailed analyses were conducted, and the level of statistical significance was set at *p* < 0.05. All statistical analyses were conducted with SPSS version 22.0 (IBM, Armonk, NY, USA).

## 3. Results

**Figure 1:**
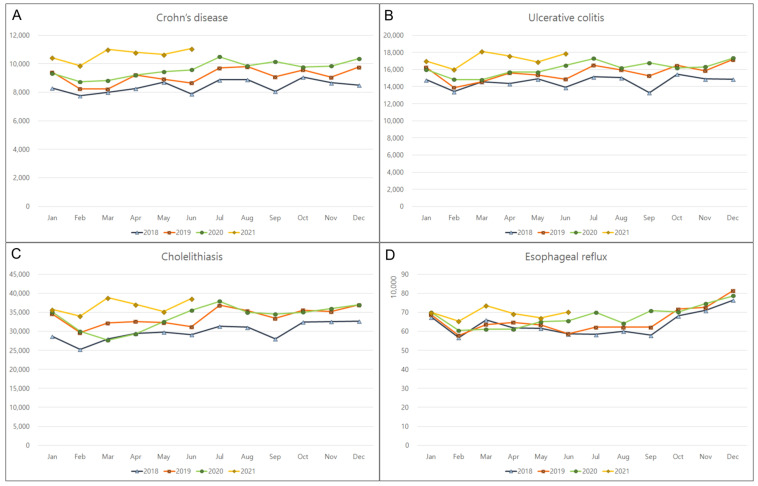
Overall increase in the monthly incidences of gastrointestinal diseases in 2018, 2019, 2020, and 2021. (**A**) Crohn’s disease, (**B**) ulcerative colitis, (**C**) cholelithiasis, and (**D**) esophageal reflux.

**Figure 2:**
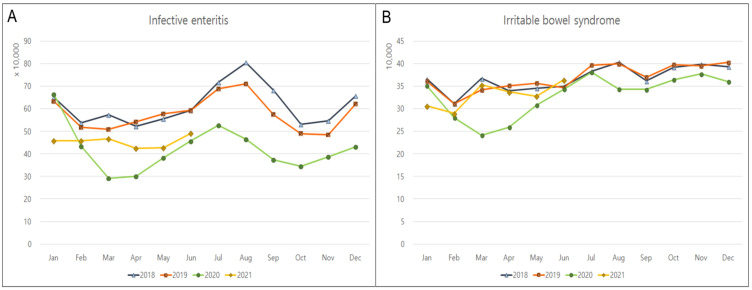
Overall decrease in the monthly incidences of gastrointestinal diseases in 2018, 2019, 2020, and 2021. (**A**) Infective enteritis and (**B**) irritable bowel syndrome.

**Figure 3:**
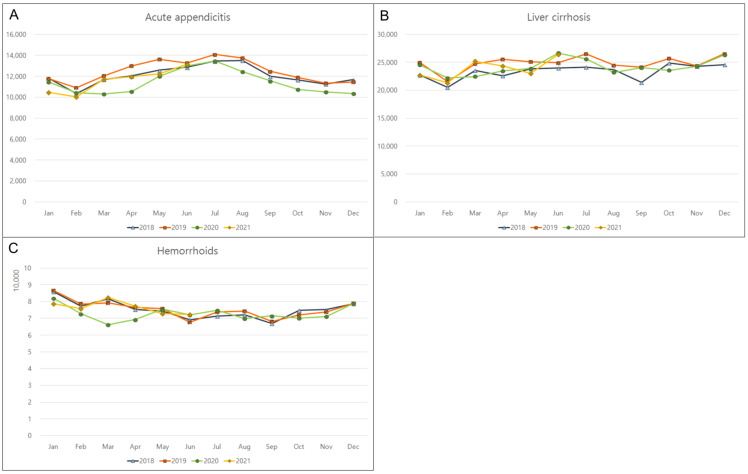
Lack of overall changes in the monthly incidences of gastrointestinal diseases in 2018, 2019, 2020, and 2021. (**A**) Acute appendicitis, (**B**) liver cirrhosis, and (**C**) hemorrhoids.

The monthly incidence rates of the top 10 GI diseases that were unrelated to COVID-19 were monitored from January 2018 to June 2021. The incidence rates of Crohn’s disease, ulcerative colitis, cholelithiasis, and esophageal reflux significantly increased by 14.6% (*p* < 0.001); 10% (*p* < 0.001), 9.7% (*p* = 0.003); and 5.8% (*p* = 0.030), respectively, during the pandemic (Table 1; Figure 1). In contrast, there was a significant decline in the incidence rates of infective enteritis (29.5%, *p* < 0.001) and irritable bowel syndrome (9%, *p* = 0.007) (Figure 2). No significant changes were observed in the incidences of pancreatitis, acute appendicitis, liver cirrhosis, or hemorrhoids during the COVID-19 pandemic (Figure 3). Monthly or seasonal variations in the incidence rates of the monitored GI diseases were unremarkable in the overall population, regardless of the COVID-19 pandemic period.

Regarding sex, fluctuations in the incidence rates throughout the COVID-19 pandemic were observed among men. There was an increase in the incidence rates of Crohn’s disease (16%), cholelithiasis (10.2%), ulcerative colitis (10%), and esophageal reflux (5%); in contrast, the incidence rates of infective enteritis and irritable bowel syndrome decreased by 30% and 10%, respectively (all *p* < 0.05, Table 2). Among women, Crohn’s disease (12%) showed the largest increase in incidence, followed by ulcerative colitis (10%), cholelithiasis (9%), and esophageal reflux (7%) (all *p* < 0.05); the incidence rates of infective enteritis and irritable bowel syndrome had significantly decreased by 28.8% (*p* < 0.001) and 8.6% (*p* = 0.004), respectively.

A subgroup analysis by age indicated that the incidence rate of Crohn’s disease in the pediatric subgroup (0–19 years old) increased during the pandemic by 16%, compared to the average rate that was reported for the prior 2 years (*p* < 0.001). In contrast, the incidence rates of infective enteritis, irritable bowel syndrome, acute appendicitis, and hemorrhoids had reduced by 42%, 25%, 21%, and 11%, respectively (all *p* < 0.05, Table 3). The monthly variation in the incidence of liver cirrhosis was lower during the COVID-19 pandemic than before (SD = 3.6 vs. 6.5, *p* = 0.010), whereas that of esophageal reflux was higher during the pandemic (SD = 4486.2 vs. 2626.0, *p* = 0.003), although the mean yearly occurrences of those diseases were relatively constant (*p* = 0.194 and *p* = 0.816, respectively).

In the adult subgroup (20–59 years old), the incidence rates of Crohn’s disease, ulcerative colitis, and cholelithiasis had increased by 13.7% (*p* < 0.001), 8.9% (*p* < 0.001), and 7% (*p* = 0.026), respectively, during the pandemic. The incidence rates of infective enteritis, irritable bowel syndrome, liver cirrhosis, hemorrhoids, and pancreatitis decreased by 24.4% (*p* < 0.001); 12.2% (*p* = 0.004); 10.4% (*p* < 0.001); 5% (*p* = 0.013); and 4% (*p* = 0.008), respectively. The monthly variation in the incidence of esophageal reflux was lower during the COVID-19 pandemic than before (SD = 27,233.5 vs. 41,252.4, *p* = 0.008).

In the older subgroup (>60 years), an increase in the incidence rates during the pandemic was observed for the following six GI diseases: Crohn’s disease (22%, *p* < 0.001); ulcerative colitis (14%, *p* < 0.001); cholelithiasis (12.1%, *p* < 0.001); esophageal reflux (10.4%, *p* = 0.001); liver cirrhosis (9%, *p* = 0.001); and pancreatitis (6%, *p* = 0.013). The incidence rate of infective enteritis had reduced by 11% during the pandemic period (*p* = 0.003).

## 4. Discussion

There is a lack of research on the differences in the national incidence rates of GI diseases before and during the COVID-19 pandemic, while accounting for the moderating effects of age and sex. Using a Korean nationwide data set, we demonstrated that the current COVID-19 pandemic led to a reconfiguration of the incidence patterns of both infectious and non-infectious GI diseases in patients without COVID-19. Over the past 2 years of the pandemic, the incidence rate of inflammatory bowel diseases (including Crohn’s disease and ulcerative colitis) showed the highest increase among all GI diseases, while that of infective enteritis showed the most significant decline; these trends were consistent, irrespective of age subgroup or sex. Our findings are applicable to non-COVID-19 GI diseases across South Korea and highlight the need for effective preventive and therapeutic public health initiatives at the national level to address anticipated changes in the burden of care, both in the recovery phase from the COVID-19 pandemic and in the post-pandemic period.

The incidence rates of inflammatory bowel diseases such as Crohn’s disease and ulcerative colitis were 14.6% and 10% higher, respectively, during the pandemic than the rates in the pre-COVID-19 period. Both of these inflammatory bowel diseases are chronic and immune-mediated conditions [17]. The finding that the incidence rates of these two inflammatory bowel diseases increased consistently across all age subgroups requires special attention, as older patients with immune-mediated diseases are particularly vulnerable to both COVID-19 and opportunistic infections and have higher morbidity and mortality rates [17]. Thus, appropriate medical care for these inflammatory bowel diseases should be specifically adapted for pediatric, adult, and older patients. With the establishment of the Realignment of the Health Care Institution Use System during the pandemic, 270 hospitals in South Korea were designated as COVID-19 protection hospitals and the remaining health care institutions were required to ensure non-COVID-19 patients’ safety and access to hospitals by separating areas for patients with respiratory illnesses from those for patients with other illnesses since 30 September 2020 [18]. Although telemedicine services were previously considered to be illegal in South Korea, the government allowed physicians to offer over-the-phone medical consultation and prescriptions temporarily, until the end of the COVID-19 outbreak. Moreover, the health care institutions and pharmacies were open as usual as per the Infectious Disease Prevention and Management Act. Therefore, the patients with chronic disease, including inflammatory bowel diseases, could use the hospitals and pharmacies without any inconvenient restrictions, so the inaccessibility of hospitals or pharmacies does not explain the increase in the incidence of inflammatory bowel diseases during the pandemic. The reason for the increase in the number of people affected by inflammatory bowel diseases during the COVID-19 pandemic is currently unclear; stress during this period might have contributed to disease deterioration [19,20]. Previous studies have linked globally stressful disasters to symptom deterioration and increased disease severity among patients with Crohn’s disease and ulcerative colitis [19,20]. The relevance of pandemic-related stress might be supported by the latest national and international demands for heightened attention to mental disorders and psychological distress during this pandemic [21,22].

We found that the incidence rates of cholelithiasis and esophageal reflux significantly increased by 9.7% and 5.8%, respectively, during the COVID-19 pandemic. The elevated incidence rate of cholelithiasis was evident in both the adult and older subgroups, while that of esophageal reflux was only identified in the older subgroup. Although cholelithiasis is a very common disease that can occur in 20% of the population in developed countries, complicated cases may require hospital admission and critical care [23]. Esophageal reflux may lead to an increased risk of both COVID-19 and aspiration pneumonia in all ages [24,25], and it might be one of the manifestations of stress affecting a wide range of gastrointestinal disorders, including inflammatory bowel disease [26]. In particular, the monthly variation in esophageal reflux was found in pediatric and adult groups, although the yearly mean disease occurrences were relatively constant. The monthly variance in esophageal reflux in children may be one of the overweight/obesity-related comorbidities that are caused by a lack of outdoor or school activities, and dietary changes such as increased snacking at home, owing to school closure [27]. Thus, medical services for these diseases may require specific care, according to age group.

Further, a significant decline in the number of cholelithiasis cases was noted in the early phase of the pandemic period; however, a subsequent increase occurred over time. This finding is in line with that of a previous study that was carried out in Germany, which reported a 20% increase in the incidence rate of cholelithiasis/cholecystitis during the COVID-19 pandemic in mid-2020, compared to that the pre-COVID-19 period in 2019 [28]. The authors suggested that postponing emergency care for patients with cholelithiasis in the beginning of the pandemic outbreak could have resulted in an increased need for care upon the subsequent loosening of the infection control measures, such as social distancing [28]. Due to the increase in the number of cholelithiasis cases, a long surgical waiting list is currently a major problem in Spanish medical institutions [29]. These observations emphasize the need for appropriate changes in health care systems to accommodate the anticipated backlog of cases requiring surgical care, as long delays before definitive treatment are likely to result in increased morbidity and mortality.

We observed a 29.5% reduction in infective enteritis cases during the COVID-19 pandemic. This finding may be ascribed to the implementation of infection control policies during the pandemic and may have implications for the incidence of other GI infectious diseases. Indeed, a previous study that was conducted in South Korea reported that viral pathogens that were responsible for GI infections drastically diminished during the pandemic period [14]. The incidence rates of infections with total viruses, norovirus, group A rotavirus, and enteric adenovirus were 32%, 40.2%, 31.8%, and 13.4% lower, respectively, during the pandemic than the rates in the 2-year period before the pandemic [14]. In South Korea, non-pharmaceutical interventions such as social distancing, online schooling, the restriction of gatherings, and personal protective measures were strictly enforced during the COVID-19 pandemic [3]. These interventions not only suppressed the spread of COVID-19, but also appeared to have a large impact on the incidence of other infectious diseases, including GI infections [5,6,7,14].

A 9% reduction in irritable bowel syndrome cases was observed in the pandemic period from that in the pre-COVID-19 period; however, the reason for this change is currently unclear, and the phenomenon is referred to as “COVID-19 irritable bowel syndrome paradox” [30]. Irritable bowel syndrome is a frequent functional GI disorder with a prevalence of 3–5% worldwide [26]. Patients may complain of repetitive abdominal pain that is accompanied with changes in the consistency or frequency of stools, without any gross peculiarity [31]. Several studies have presented strong evidence that irritable bowel syndrome is a stress-sensitive illness; the primary effects of psychosocial stress on gut physiology involve increases in visceral hypersensitivity and mucosal permeability, changes in gut motility and secretions, and negative effects on gut microbiota [26,32]. The sudden arrival of the COVID-19 pandemic likely acted as a major stressor for patients with irritable bowel syndrome [26]. Unexpectedly, our survey showed a paradoxical decline in irritable bowel syndrome cases during the COVID-19 pandemic, instead of symptom deterioration [30]. Lockdowns allowed easy access to toilets at home, and the subsequent adoption of flexible work and school schedules in Singapore, Japan, and France resulted in a decrease in illnesses, as well as improvements in sleep and exercise, reduced burnout, an improved sense of well-being, and a reduction in commute-related stress [30,31,33]. These factors may have contributed to the decrease in symptom severity among patients with irritable bowel syndrome, which might have reduced their need for professional medical care [31].

No significant changes in yearly average incidences of pancreatitis, acute appendicitis, liver cirrhosis, or hemorrhoids were observed in the overall population between the pre-COVID-19 and pandemic periods, suggesting that these diseases might not have been severely affected by the lifestyle changes that were made during the pandemic. Pancreatitis and liver cirrhosis are likely related to existing alcohol-use disorder, because alcohol-related pancreatic or liver disease often occurs after years of regular, heavy alcohol use [15]. Since those diseases are relatively common, sometimes requiring emergency surgery or critical care [15,34,35], the similar rates of medical visits for those diseases between two periods may be related to the stability of medical systems. In addition, there were no monthly or seasonal variations in GI diseases in the overall population when comparing the corresponding months before and during the COVID-19 pandemic, except for some diseases in different age groups. Hence, either changes in disease incidence rates were evenly distributed across all seasons, or there were no seasonal variations in disease incidence rates.

This study has several limitations. First, the results might not be applicable to other countries due to discrepancies in the severity of the COVID-19 pandemic in different countries [36,37]. Second, this study was based on health claims data that categorized diseases using diagnostic codes; therefore, undiagnosed or subclinical diseases could not be accounted for. Third, specific data on the differences in presentations between primary care and hospital visits, which might indicate disease severity, were not collected. In addition, we were unable to reconfirm the accuracy of the diagnoses, as we did not have access to laboratory test results. In order to minimize potential bias due to disease misclassification, we analyzed data of 2 years (2018 and 2019) before the COVID-19 pandemic period. Fourth, possible bias due to multiple comparisons could not be excluded, as we analyzed 10 GI diseases. Nonetheless, the main strength of this study is that it collected data from a centralized nationwide healthcare database that included every citizen that attended a primary care or hospital clinic. Therefore, our results may offer valuable information for understanding the main changes in GI diseases and the possible underlying causes during the pandemic crisis in the South Korean context.

## 5. Conclusions

The COVID-19 pandemic era has altered the incidence patterns of certain GI diseases, and this may foreshadow an increased burden of those diseases in the aftermath of the pandemic. The unprecedented changes highlight the need for the restructuring of healthcare systems at a national level to address anticipated changes in the burden of care, both in the recovery phase and the post-pandemic period.

## Figures and Tables

**Table 1:** Differences in the means and standard deviations of incidences of diseases before and during COVID-19.

Diseases	Before COVID-19		During COVID-19		*p*-Values of Difference	
	Mean	SD	Mean	SD	Mean	Variance ^†^
Cholelithiasis	31,908.2	2980.4	34,999.7	3053.7	0.003 *	0.842
Pancreatitis	9376.2	487.7	9397.0	562.6	0.786	0.952
Infective enteritis	592,974.9	86,207.8	418,206.6	66,354.2	<0.001 *	0.117
Crohn’s disease	8799.0	595.7	10,087.6	644.9	<0.001 *	0.268
Ulcerative colitis	15,125.1	946.1	16,638.8	889.8	<0.001 *	0.501
Acute appendicitis	12,173.0	1016.7	11,537.4	1127.0	0.100	0.506
Liver cirrhosis	24,059.7	1490.0	24,171.6	1535.7	0.816	0.559
Esophageal reflux	647,717.8	62,784.3	685,385.2	47,978.9	0.030 *	0.141
Hemorrhoids	75,509.2	5076.9	73,697.4	4252.5	0.300	0.988
Irritable bowel syndrome	364,276.6	31,985.2	331,089.3	40,512.7	0.007 *	0.499

* Mann–Whitney *U* test, significance at <0.05. ^†^ Levene’s test in non-parametric data, significance at <0.05.

**Table 2:** Sex-based differences in the means and standard deviations of incidences of diseases before and during COVID-19.

Diseases	Before COVID-19		During COVID-19		*p*-Values of Difference	
	Mean	SD	Mean	SD	Mean	Variance ^†^
Men						
Cholelithiasis	15,126.3	1399.4	16,671.3	1336.0	0.002 *	0.901
Pancreatitis	6460.2	326.1	6424.1	352.6	0.698	0.990
Infective enteritis	280,916.2	42,954.1	195,956.6	32,499.2	<0.001 *	0.109
Crohn’s disease	6170.8	426.8	7138.8	468.1	<0.001 *	0.187
Ulcerative colitis	9028.5	554.6	9952.4	508.6	<0.001 *	0.282
Acute appendicitis	6292.9	508.5	5951.8	554.3	0.070	0.342
Liver cirrhosis	14,234.9	830.9	14,091.3	858.3	0.338	0.672
Esophageal reflux	274,281.2	29,925.5	287,298.8	22,815.4	0.049 *	0.141
Hemorrhoids	39,706.4	2771.5	38,089.9	2122.7	0.055	0.674
Irritable bowel syndrome	170,369.7	16,209.7	153,845.9	19,914.3	0.011 *	0.866
Women						
Cholelithiasis	16,781.9	1598.0	18,328.4	1739.5	0.005 *	0.942
Pancreatitis	2935.6	173.7	2972.9	216.8	0.453	0.211
Infective enteritis	312,058.7	43,398.8	222,250.1	33,911.1	<0.001 *	0.103
Crohn’s disease	2628.3	176.0	2948.8	182.7	<0.001 *	0.459
Ulcerative colitis	6096.6	396.8	6686.4	391.3	<0.001 *	0.579
Acute appendicitis	5880.1	518.6	5585.6	580.9	0.178	0.499
Liver cirrhosis	9824.8	667.1	10,080.3	699.2	0.468	0.550
Esophageal reflux	373,436.5	33,381.3	398,086.4	25,737.6	0.013 *	0.276
Hemorrhoids	35,802.8	2341.0	35,607.6	2195.4	0.816	0.878
Irritable bowel syndrome	193,907.0	16,213.5	177,243.3	20,934.0	0.004 *	0.451

* Mann–Whitney *U* test, significance at <0.05. ^†^ Levene’s test in non-parametric data, significance at <0.05.

**Table 3:** Age-based differences in the means and standard deviations of incidences of diseases before and during COVID-19.

Diseases	Before COVID-19		During COVID-19		*p*-Values of Difference	
	Mean	SD	Mean	SD	Mean	Variance
Age 0–19 years old						
Cholelithiasis	126.0	17.4	129.3	18.3	0.364	0.469
Pancreatitis	154.9	24.2	150.4	24.4	0.907	0.140
Infective enteritis	253,267.9	43,009.3	148,682.7	36,095.9	<0.001 *	0.076
Crohn’s disease	1198.3	72.2	1391.4	139.5	<0.001 *	0.905
Ulcerative colitis	510.6	40.3	506.8	34.0	0.907	0.923
Acute appendicitis	2668.0	334.7	2128.2	321.7	<0.001 *	0.211
Liver cirrhosis	21.9	6.5	20.1	3.6	0.194	0.010 ^†^
Esophageal reflux	18,068.5	2626.0	18,186.0	4486.2	0.816	0.003 ^†^
Hemorrhoids	2165.0	283.3	1941.1	166.7	0.004 *	0.895
Irritable bowel syndrome	59,571.1	9637.7	44,838.4	9480.1	<0.001 *	0.173
Age 20–59 years old						
Cholelithiasis	14,874.7	1467.3	15,915.9	1463.0	0.026 *	0.754
Pancreatitis	5609.0	257.6	5393.8	278.6	0.008 *	0.680
Infective enteritis	249,570.0	40,599.1	189,414.1	29,746.8	<0.001 *	0.428
Crohn’s disease	6928.2	491.9	7876.1	456.5	<0.001 *	0.272
Ulcerative colitis	10,469.3	628.4	11,397.7	578.1	<0.001 *	0.651
Acute appendicitis	7431.3	626.9	7214.0	664.0	0.319	0.183
Liver cirrhosis	10,709.2	537.2	9589.1	678.8	<0.001 *	0.296
Esophageal reflux	353,522.5	41,252.4	362,350.3	27,233.5	0.147	0.008 ^†^
Hemorrhoids	54,427.3	3560.4	51,725.9	2417.1	0.013 *	0.510
Irritable bowel syndrome	163,525.9	18,245.6	143,566.3	20,468.9	0.004 *	0.861
Age 60+ years old						
Cholelithiasis	16,934.4	1563.0	18,987.3	1649.2	<0.001 *	0.848
Pancreatitis	3639.8	257.5	3861.1	289.1	0.013 *	0.365
Infective enteritis	90,246.6	8755.6	80,201.4	8146.2	0.003 *	0.575
Crohn’s disease	680.1	67.3	829.5	78.0	<0.001 *	0.191
Ulcerative colitis	4156.8	313.8	4748.4	312.2	<0.001 *	0.163
Acute appendicitis	2086.7	149.7	2209.4	203.7	0.076	0.312
Liver cirrhosis	13,350.0	1051.1	14,581.4	1014.7	0.001 *	0.701
Esophageal reflux	276,550.2	24,140.3	305,290.8	21,752.4	0.001 *	0.405
Hemorrhoids	19,012.7	1777.7	20,122.5	2018.1	0.120	0.916
Irritable bowel syndrome	141,340.6	9617.7	142,834.1	13,519.3	0.365	0.100

* Mann–Whitney *U* test, significance at <0.05. ^†^ Levene’s test in non-parametric data, significance at <0.05.

## Data Availability

All data are available from the database of the National Health Insurance Sharing Service (NHISS) https://nhiss.nhis.or.kr/ (accessed on 31 August 2021). The NHISS grants researchers access to its data if they agree to follow the research ethics at a cost. Those seeking access to the data in this article can download the data from the website after agreeing to follow the research ethics.
